# Autonomous Binarized Focal Loss Enhanced Model Compression Design Using Tensor Train Decomposition

**DOI:** 10.3390/mi13101738

**Published:** 2022-10-14

**Authors:** Mingshuo Liu, Shiyi Luo, Kevin Han, Ronald F. DeMara, Yu Bai

**Affiliations:** 1 Electrical and Computer Engineering Department, College of Engineering and Computer Science, California State University, 800 N State College Blvd, Fullerton, CA 92831, USA; 2 Department of Electrical and Computer Engineering, College of Engineering and Computer Science, University of Central Florida, 4000 Central Florida Blvd, Orlando, FL 32816, USA

**Keywords:** tensor decomposition, focal loss, embedded hardware

## Abstract

Deep learning methods have exhibited the great capacity to process object detection tasks, offering a practical and viable approach in many applications. When researchers have advanced deep learning models to improve their performance, the model derived from the algorithmic improvement may itself require complementary increases in computational and power demands. Recently, model compression and pruning techniques have received more attention to promote the wide employment of the DNN model. Although these techniques have achieved a remarkable performance, the class imbalance issue during the mode compression process does not vanish. This paper exploits the Autonomous Binarized Focal Loss Enhanced Model Compression (ABFLMC) model to address the issue. Additionally, our proposed ABFLMC can automatically receive the dynamic difficulty term during the training process to improve performance and reduce complexity. A novel hardware architecture is proposed to accelerate inference. Our experimental results show that the ABFLMC can achieve higher accuracy, faster speed, and smaller model size.

## 1. Introduction

The current state-of-the-art object detection network using Deep Learning conducts a competition between various models due to their incredible feats in the field. Deep learning techniques are widely adopted in various applications, such as self-driving UAVs, Water Quality Prediction [1], autonomous robotics, and robot vision. It is clearly seen that these tasks demand a Deep Learning technology with high accuracy, smaller size, and low latency model running on mobile electronic devices.

Among various deep learning techniques in the object detection field based on Convolutional Neural Networks (CNNs), there are two approaches that can be summarized in the past decades [2]: one stage approach [3] and two-stage approach [4]. Although the latter method achieves a remarkable accuracy performance on object detection benchmarks COCO [5], the models suffer from a longer execution time. In contrast, a one-stage detector runs faster, suffering from poorer accuracy. Although these one- and two-stage approaches have demonstrated powerful capabilities in many applications, the previous research paper [6] has indicated that class imbalance has been identified as a key effect of this performance gap. Two-stage approaches can immune this imbalance problem as they remove the background before identifying the objects [7]. In addition, these approaches use fixed background ratio [4] or online hardware example mining [8] to balance the training data in the second stage. In contrast, the one-stage detector faces more challenges since it needs to learn the difference between foreground classes and possible scenery. Thus, the two approaches mentioned above cannot be applied directly to a one-stage detector.

Furthermore, although class imbalance has been addressed in the R-CNN series detectors using a two-stage cascade and sampling heuristics [6], the one-stage detector must process large candidate locations in an image. For example, in practice, the detector may receive ∼100K candidate locations in the image. Thus, it is inefficient during the training procedure when the samples are dominated by easily classified background examples.

Recent work has been proposed to employ α balancing to the loss function to distinguish classes and backgrounds. This method is intended to improve the detector to avoid imbalance classification. After this first attempt toward a dynamic loss function, RetinaNet [6] has added a data dynamic to the loss function, named Focal Loss. The probability-based difficulty for the correct class is calculated differently in each classification. In this work, a hyper-parameter γ is added to control the loss function. However, different neural network models may require different hyper-parameters to obtain the best accuracy. In our earlier work [9,10], we have shown that the class imbalance issue gets worse during model compression, and we have demonstrated that the Focal Loss can improve the model accuracy within imbalanced data. However, the proposed Focal Loss is difficult to obtain preliminary information and fine-tune the parameters.

**Limitations of Existing Imbalance Issue on Model Compression:** Although the model compression approach plays an important role in developing efficient deep leanings [11,12], the state-of-the-art model compression methods still suffer from a class imbalance issue due to three challenging limitations. First, the existing works only focus on the model size and accuracy of the model as its target objectives without considering the issue of class imbalance. Second, although TT-decomposition achieves a remarkable result in compressing the model without significant accuracy drops, the added more tensor cores create a complex parameter setting when we apply focal loss technology directly to the model compression algorithm. Third, current focal loss methods have been implemented only on software platforms. Thus, such focal loss methods can only achieve suboptimization and lead to performance drop on model compression when we employ the resource constraint hardware.

In this paper, a novel approach constructing an autonomous focal loss algorithm that performs an efficient loss function for class imbalance issues during model compression is presented. We first attempt to address the imbalance issue during the model compression. In addition, a high-performance hardware accelerator is developed in this paper. Specifically, the main technical contributions are as follows:

- In this work, we first attempt to consider the class imbalance issue during compression of the tensor train-based model. The proposed ABFLMC algorithm can be used for the tensor train decomposition method to overcome the class imbalance issue.
- At the algorithm level, the proposed ABFLMC algorithm is designated by considering the characteristics of the tensor train decomposition to reduce the complexity and increase the performance. Consequently, the proposed framework can automatically search for the best parameters for overcoming the imbalance issue during the training process.
- At the hardware design level, the architecture of the proposed ABFLMC algorithm has been developed to maximize parallelism and processing throughput. Thus, the proposed ABFLMC algorithm can achieve an optimized solution on the resource constraint hardware.

The remainder of this paper is organized as follows: In Section 2, we introduce the related background in object detection, focal Loss, and tensor train decomposition. In Section 3, we demonstrate a novel algorithm for optimizing autonomous focal loss models (ABFLMC) to overcome unbalanced issues during model compression. Section 4 discusses the hardware design of the ABFLMC model. Section 5 presents experimental results in both software and hardware. Finally, Section 6 concludes the manuscript.

## 2. Related Work

### 2.1. Object Detection

Object detection is a fundamental task in the field of computer vision. The purpose of an object detector is to classify and localize all objects in an image or video. Object detectors are designed to extract hand-crafted features [13,14], which are widely employed in various branches like self-driving cars. With the increasing size of datasets, object detectors become larger and larger, leading to decreased speed and accuracy. Thus, much research has been done to improve object detectors. Generally, object detectors can be summarized into two categories according to the structure: one-stage and two-stage detectors.

**Two-Stage Detectors**: In paper [4], Girshick et al. propose a region-based convolutional neural network (R-CNN) using AlexNet [15] as the backbone. This framework employs the region proposal module to extract features. After the feature is extracted, it is classified by class-specific support vector machines (SVMs) to obtain scores. Although it achieves good performance, the longer execution time to detect an image is a drawback. Some research efforts such as Fast R-CNN [16] have been proposed to overcome this problem. The Fast R-CNN is trained with an end-to-end architecture, and a multitask loss, which is very simple for the training and can improve operation speed and forecast accuracy. After researchers constantly strive for excellence, a novel model that combines Fast R-CNN and a region proposal network (RPN) is proposed [7]. This faster R-CNN can learn and generate better region proposals using CNN used in the region proposal module, leading to improved accuracy. Later, an R-FCN [17] combined with faster R-CNN and FCN is presented to reduce model training time. The proposed R-FCN can improve the 2.5–20× speed compared to Faster R-CNN. However, since it uses ResNet101, the model is difficult to implement on devices with limited resources. Recently, DetectoRS [18] employing Recursive Feature Pyramid (RFP), Atrous Spatial Pyramid Pooling (ASPP), and Switchable Atrous Convolution (SAC) is proposed. This work builds a switch system to control the rate of convolution. Consequently, it can improve the detection of multi-scale objects. Although the DetectoRS have improved the model performance, it is still ineffective in real-time tasks due to its complexity.

**One-Stage Detectors**: Compared with two-stage detectors, the one-stage detectors exchange object detector tasks from classification to a regression. You Only Look Once (YOLO) [19] utilizes multiple smaller convolutional networks in a cascading way to predict the image directly with a bounding box. An image is divided into N×N parts, and each of the parts must predict multiple bounding boxes. In this way, the YOLO model achieves a significant improvement in both running speed and accuracy. Following the YOLO Model, Single Shot MultiBox Detector (SSD) [3] is proposed. It can balance the speed and accuracy of real-time detection tasks. Its accuracy achieves similar performance to a two-stage detector Faster R-CNN. Later, a variant of the YOLO network-YOLOv2 (YOLO9000) [20] is presented. It uses Darknet-19 [21] as the backbone architecture and uses multiple efficient techniques such as Batch Normalization [22], WordTree [23] to improve efficiency. The fully-connected layer is removed to enhance the inference speed. YOLOv2 becomes “better, faster, and stronger”. Within the YOLO series, Yolov4 [24] is proposed for object detection and achieves a new record speed (65FPS).

Many innovations are implemented within the framework of YOLOv4. Specifically, the “Bag of Freebies” includes class label smoothing, data augmentation, and the Cross mini-Batch Normalization (CmBN) algorithm that collects the statistics between the mini-batches, these technologies are efficient to improve model performance without increasing the inference time. However, the "Bag of Special" with CSP (Cross-stage partial connection), SPP module, and PAN neck still leads to the inference time rise [25]. The latest variant of YOLO YOLOv5 [26] has been proposed to improve YOLOV4. However, in general, these object detectors are not feasible on resource-constrained devices such as non-GPU laptops and mobile devices. Current research efforts, including MobileNet-SSDLite [27], develop lightweight depth-wise convolutions to extract features and use the delinearized module in low-dimensional layers. Both the execution time of the operation and the size of the model are decreased while maintaining the same accuracy. Following this work, YOLO-LITE [28] and Tiny-DSOD [29] aim to create a faster, smaller, and high-performed model enhancing the accessibility that makes real-time detection model deployed on all devices. Moreover, many compression algorithms like pruning and tensor-train decomposition are introduced to improve computational efficiency. YOLObile [30] presents a novel block-punched pruning scheme that exhibits a high accuracy on mobiles and embedded devices.

**Class Imbalance Issue**: In the paper [6], Tsung-Yi Lin et al. state that the object detection models encounter the class imbalance problem while training. There are $10^{4}$–$10^{5}$ potential samples per image; however, a small amount of them comprise the desired objects. The imbalance would make training inefficient and disturb the training process because of many easy negative samples. To address this issue, they came up with the focal loss. This focal loss can naturally deal with class imbalance without the traditional sampling method by applying the balance factor and modulating the factor into the loss function.

### 2.2. Tensor Decomposition Methods

The tensors are important for an efficient computation since a large number of dimensions of tensors demands an intensive memory. The number increases exponentially with the number of dimensions. Over the last decades, Many notable tensor decomposition approaches are proposed, including Tucker decomposition [31], the canonical polyadic decomposition (CPD) [32,33,34], parallel factor (PARAFAC2) [35], CTSVD-QR [36] and tensor train decomposition (TT decomposition) [10,37,38]. Among these approaches, TT decomposition is an impressive method with some benefits, such as efficient tensor representation (TT format) dedicated to reducing memory storage and flexible and high-efficiency reasoning logic with singular value decomposition. Moreover, experimental results in [39] show that TT decomposition can be applied to fully connected (FC) layers, resulting in a significant reduction in the number of parameters with a tiny drop in accuracy. After that, Novikov1 et al. adopt the TT decomposition to tensorflow for its easier utilization [40]. Subsequently, Garipov et al. [41] propose a novel method that applies TT decomposition to both convolutional layers and fully-connected layers. Therefore, the TT decomposition approach has become a very promising model compression tool.

In detail, TT decomposition is capable of factorizing a *d*-dimensional tensor with the size of $n_{1}\times n_{2}\times \ldots \times n_{d}$ into several tensor cores with the size of $r_{k-1}\times n_{k}\times r_{k}$. To expound the concept of TT decomposition, we utilize the naming convention from Garipov et al. [41]. A tensor is defined as $A\in R^{n_{1}\times n_{2}\times \ldots \times n_{d}}$. The TT-representation of the tensor A is a set of TT-cores $G_{k}\left[j_{k}\right]\in R^{r_{k-1}\times r_{k}}$, where $j_{k}\in \left[1,n_{k}\right],k=1,2,\ldots ,d$. Therefore, $A(j_{1},j_{2},\ldots ,j_{d})$ as the element of the tensor A can be decomposed as:

(1) $$A\left(j_{1},j_{2},\ldots ,j_{d}\right)=G_{1}\left[j_{1}\right]G_{2}\left[j_{2}\right]\ldots G_{d}\left[j_{d}\right]$$

where $r_{k}$ is the rank value, and $r_{0}=r_{d}=1$ are adopted to ensure the feasibility of TT decomposition. After the decomposition of the TT, the number of parameters of the tensor A is $\sum_{k=1}^{d}r_{k-1}n_{k}r_{k}$, which is significantly less than the original size $\prod_{k=1}^{d}n_{k}$. Therefore, the compression ratio can be defined as $\prod_{k=1}^{d}n_{k}/\sum_{k=1}^{d}r_{k-1}n_{k}r_{k}$, and choosing the balanced value of $r_{k}$ is significant in maximizing the compression ratio.

## 3. Autonomous Binarized Focal Loss Enhanced Model Compression Algorithm (ABFLMC)

### 3.1. TT-Convolutional Layer in the Model YOLOV5

**Figure 1:**
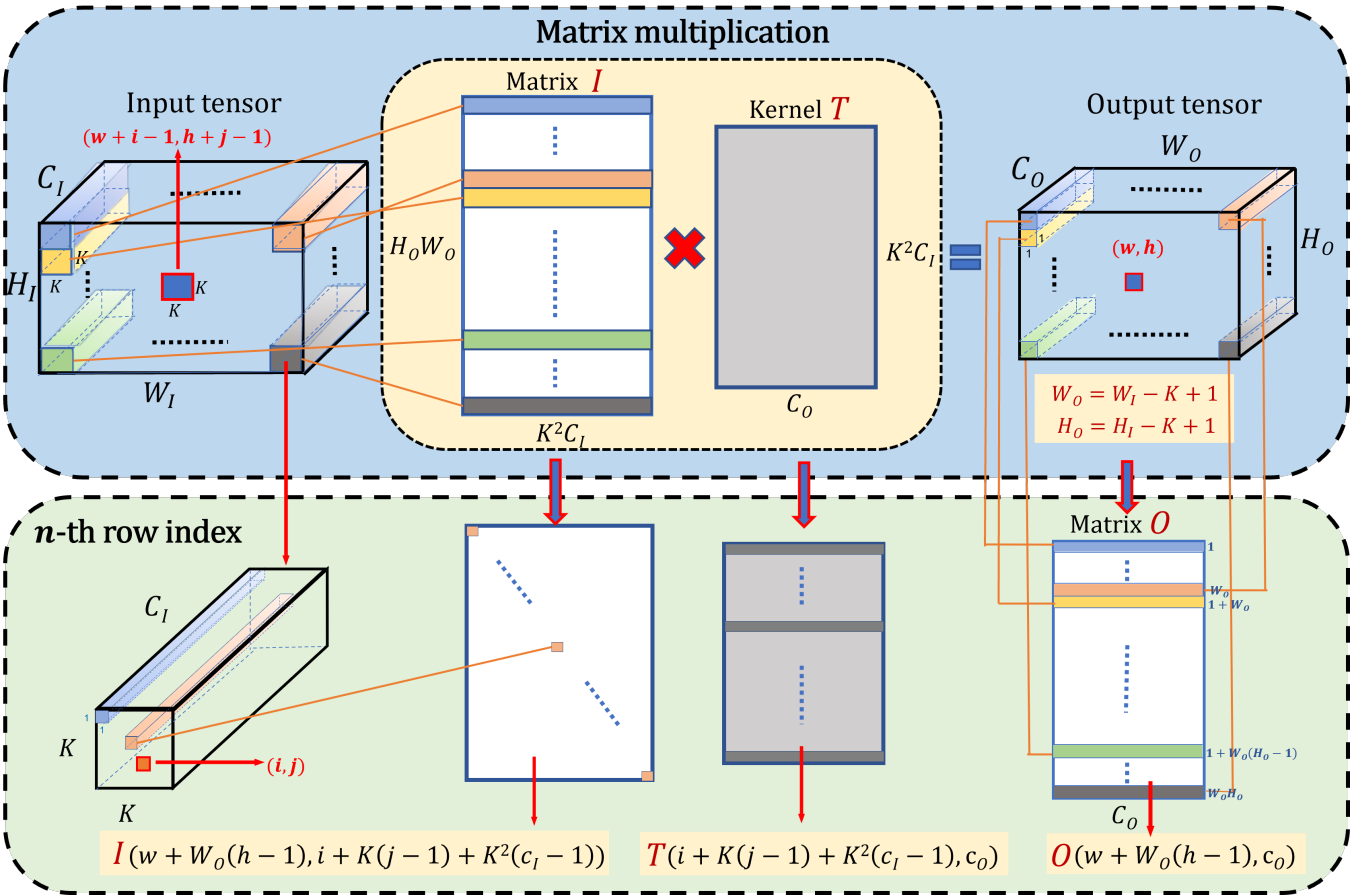
Tensor Train convolutional process: The top shows a convolutional layer can be reformulate to a matrix-by-matrix multiplication O=I×T, the bottom introduces how to calculate the *n*-th row of the matrices that correspond to the $K\times K\times C_{I}$ patch of the input tensor as Equation (2) illustrates.

Garipov et al. [41] point out that using the TT-decomposition to factorize the convolutional kernel into the product of several low-rank matrices directly has the limitation on the convolutional layer. To address this drawback, a new decomposition is introduced that can be applied to the convolutional and fully connected layers. For a convolutional layer, assume that the input tensor I has the size $W_{I}\times H_{I}\times C_{I}$, the output tensor $O\in R^{W_{O}\times H_{O}\times C_{O}}$, and the kernel tensor T is a 4-dimensional tensor with the size $K\times K\times C_{I}\times C_{O}$. Now, we can easily write the formula for the operation of a YOLOV5 convolutional layer as: $O\left(w,h,c_{O}\right)=\sum_{i=1}^{K}\sum_{j=1}^{K}\sum_{c_{I}=1}^{C_{I}}T\left(i,j,c_{I},c_{O}\right)I\left(w+i-1,h+j-1,c_{I}\right)$. Figure 1 shows the Tensor Train process and the element shift process. The convolutional formula can also be calculated as several matrix multiplications O=I×T. The top in Figure 1 shows how the input tensor is decomposed into several matrix multiplications. The purpose of the following derivation is to express the relationship between the input tensor, output tensor, and the kernel tensor via introducing how the *n*-th row of the input matrix is used to compute the *n*-th row of the output of the matrix multiplication. First, we transfer the input tensor $I\in R^{W_{I}\times H_{I}\times C_{I}}$ and the output tensor $O\in R^{H_{O}\times W_{O}\times C_{O}}$ to matrices. To analyze the process, we define a patch of the input tensor within size $K\times K\times C_{I}$ and a patch of the output tensor is $1\times 1\times C_{O}$, so we easily obtain the connection $H_{O}=H_{I}-K+1$ and $W_{O}=W_{I}-K+1$. Now, we remodel the output tensor O into a matrix O: $O\left(w,h,c_{O}\right)=O\left(w+W_{O}\left(h-1\right),c_{O}\right)$, where $w+W_{O}\left(h-1\right)$ represents the position of the patch in the plane $H_{O}\times W_{O}$ of the output tensor that is transferred to the height of the output matrix O. Furthermore, $c_{O}$ is the width of matrix O, where $w\in (1,\ldots ,W_{O})$ and $h\in (1,\ldots ,H_{O})$. In this way, the 3-dimensional tensor can be reshaped into a 2-dimensional matrix. Therefore, the input tensor I also can be resized as follows:

(2) $$\begin{array}{cc} \; & I(w+i-1,h+j-1,c_{I})=\\ & I(w+W_{O}\left(h-1\right),i+K\left(j-1\right)+K^{2}\left(c_{I}-1\right))\\ \; & where\;i,j\in (1,\ldots ,K) \end{array}$$

Then, we can obtain the kernel matrix of size $K^{2}C_{I}\times C_{O}$ from the kernel tensor T: $T\left(i,j,c_{I},c_{O}\right)=T\left(i+K\left(j-1\right)+K^{2}\left(c_{I}-1\right),c_{O}\right)$.

In the following work, the TT format is introduced to decompose the matrix using the coincidence of the TT decomposition and the low-rank decomposition. More specifically, we have reshaped the tensor into the matrix before, then we transfer the matrix to a more compact tensor, and TT-decomposition is applied on the new tensor to obtain the matrix format TT. Let us assume a matrix $X\in R^{M\times N}$, $M=\prod_{a=1}^{d}m_{a}$ and $N=\prod_{a=1}^{d}n_{a}$. Two objective functions can be constructed to shape the matrix X into the tensor $X\in R^{n_{1}m_{1}\times n_{2}m_{2}\times \ldots \times n_{a}m_{a}}$ in the following way:

(3) $$\begin{array}{cc} \; & F\left(i\right)=[f_{1}\left(i\right),\ldots f_{a}\left(i\right),\ldots ,f_{d}\left(i\right)]\\ \; & G\left(j\right)=[g_{1}\left(j\right),\ldots g_{a}\left(j\right),\ldots ,g_{d}\left(j\right)]\\ \; & where\;f_{a}\left(i\right)\in \left(1,\ldots ,m_{a}\right)\;\;g_{a}\left(j\right)\in \left(1,\ldots ,n_{a}\right)\\ \; & \;\;\;\;\;\;\;a\in (1,\ldots ,d)\;i\in (1,\ldots ,M)\;j\in (1,\ldots ,N) \end{array}$$

Sequentially, using the TT-format to represent the elements, the X(i,j) is defined as:

(4) $$\begin{array}{cc} \; & \;\;\;\;\;\;\;\;\;\;\;\;\;\;\;\;\;\;\;\;\;\;\;\;\;\;\;\;\;\;\;\;\;X(i,j)\\ \; & =X(\left(f_{1}\left(i\right),g_{1}\left(j\right)\right),\ldots ,\left(f_{a}\left(i\right),g_{a}\left(j\right)\right),\ldots ,\left(f_{d}\left(i\right),g_{d}\left(j\right)\right))\\ \; & =G_{1}[\left(f_{1}\left(i\right),g_{1}\left(j\right)\right]\ldots G_{a}[\left(f_{a}\left(i\right),g_{a}\left(j\right)\right]\ldots G_{d}[\left(f_{d}\left(i\right),g_{d}\left(j\right)\right] \end{array}$$

According to the TT-representation, it can reshape these matrices I,O, and T into new tensors $\hat{I},\hat{O}$, and $\hat{T}$. Herein, we firstly define $C_{I}=\prod_{a=1}^{d}C_{I_{a}}$ and $C_{O}=\prod_{a=1}^{d}C_{O_{a}}$, the output matrix O can be reshaped into a new tensor $\hat{O}$ with size $W_{O}\times H_{O}\times C_{O_{1}}\times \ldots \times C_{O_{a}}\ldots \times C_{O_{d}}$, then the new input tensor is $\hat{I}$ of size $\left(W_{I}+K-1\right)\times \left(H_{I}+K-1\right)\times C_{I_{1}}\times \ldots \times C_{I_{a}}\ldots \times C_{I_{d}}$. For the kernel tensor, we deduce the formula according to Equation (4) as:

(5) $$\begin{array}{cc} \; & T(i+K\left(j-1\right)+K^{2}\left(\hat{c_{I}}-1\right),\hat{c_{O}})\\ \; & =\hat{T}\left(\left(i+K\left(j-1\right),1\right),\left(c_{I_{1}},c_{O_{1}}\right),\ldots ,\left(c_{I_{a}},c_{O_{a}}\right),\ldots ,\left(c_{I_{d}},c_{O_{d}}\right)\right)\\ \; & \;\;\;\;where\;\hat{c_{I}}=c_{I_{1}}+\sum_{i=2}^{d}\left(c_{I_{i}}-1\right)\prod_{j=1}^{i-1}c_{I_{j}}\\ \; & \;\;\;\;\;\;\;\;\;\;\;\;\;\hat{c_{O}}=c_{O_{1}}+\sum_{i=2}^{d}\left(c_{O_{i}}-1\right)\prod_{j=1}^{i-1}c_{O_{j}} \end{array}$$

Then we use TT-decomposition to factorize the kernel tensor as:

(6) $$\begin{array}{cc} \; & T(i+K\left(j-1\right)+K^{2}\left(\hat{c_{I}}-1\right),\hat{c_{O}})\\ \; & =\hat{G_{0}}\left[i+K\left(j-1\right),1\right]G_{1}\left[c_{I_{1}},c_{O_{1}}\right]\ldots G_{a}\left[c_{I_{a}},c_{O_{a}}\right]\ldots G_{d}\left[c_{I_{d}},c_{O_{d}}\right] \end{array}$$

where $\hat{G_{0}}$ is the tensor core related to the convolution kernel and $G_{1}$ to $G_{d}$ are the regular tensor cores as we mentioned above. Finally, the convolution layer can be rewritten using the TT-format:

(7) $$\begin{array}{cc} \; & \hat{O}\left(w,h,c_{O_{1}},\ldots ,c_{O_{a}},\ldots ,c_{O_{d}}\right)\\ \; & =\sum_{i=1}^{K}\sum_{j=1}^{K}\sum_{c_{I_{1}},..,c_{I_{a}},..,c_{I_{d}}}\hat{I}\left(w+i-1,h+j-1,c_{I_{1}},\ldots ,c_{I_{a}},\ldots ,c_{I_{d}}\right)\\ \; & \hat{G_{0}}\left[i+K\left(j-1\right),1\right]G_{1}\left[c_{I_{1}},c_{O_{1}}\right]\ldots G_{a}\left[c_{I_{a}},c_{O_{a}}\right]\ldots G_{d}\left[c_{I_{d}},c_{O_{d}}\right] \end{array}$$

### 3.2. Design of ABFLMC

**Figure 2:**
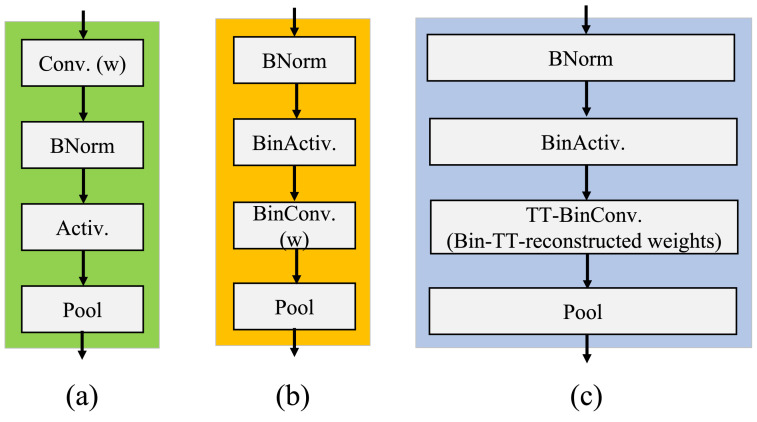
(**a**) Conventional CNN blocks, (**b**) XNOR-network blocks, (**c**) ABFLMC blocks.

The use of compact data types, such as 1-bit representations, is a current trend to enhance the effectiveness of deep neural networks. A Binary Neural Network (BNN) is a Convolutional Neural Network (CNN) of low accuracy with binarized activations and weights. BNNs normally include several layers, such as the convolutional layer, fully connected layer, pooling layer, and batch normalization layer. As shown in Figure 2a,b, an XNOR network has distinct functional blocks than CNNs. Generally, the convolutional layer, the batch normalization layer, the activation layer, and the pooling layer are just examples of the various functional layers that make up a typical neural network. The input tensor of the batch normalization layer can be normalized by computing its mean and variance. An element-wise nonlinear function (e.g., Sigmoid, ReLU) is applied to the activation layer. The pooling layer uses several pooling techniques (e.g., max, min, and average). Compared to CNN, the functional layers of the BNN are arranged differently in Figure 2b. When receiving a binarized input batch, the pooling layer suffers a significant information loss. For instance, the input batch of the min-pooling layer accepts the binarization and returns it with the majority of its members equal to −1. Thus, the grouping directly connects the convolutional layer (BinConv) to overcome the critical issue of considerable information loss. We normalize the input prior to binarization in the BNN to address the information loss problem brought on by binarization. In this case, the normalization process is efficient in increasing the model precision by forcing the input to hold a zero mean, whose threshold range shrinks to zero, resulting in a reduced binarization error. To calculate the sign(I), the binary activation layer (BinActiv) is utilized.

To combine Tensor Train Decomposition method with the binary convolution layer, we designed a structure shown in Figure 2c. The Tensor Train cores could be stored in binary format with the scaling factors for each, which is the same as in the XNOR network. After the TT reconstruction of the real weight, as demonstrated above, we would binarize it and use another scaling factor α along the real weight to perform the binary convolution. Thus, the TT-format binary convolution can be rewritten in short as:

(8) $$\begin{array}{cc} \; & \alpha_{G}B\left(\alpha_{0}\alpha_{1}\ldots \alpha_{a}\ldots \alpha_{d}B\left(\hat{G_{0}}G_{1}\ldots G_{a}\ldots G_{d}\right)\right)=\alpha_{G}B\left(T\right)*I\approx \sum \alpha_{G_{f}}\left(B\left(T_{f}\right)⊕I\right) \end{array}$$

where B denotes the element wise weight binarization, $\alpha_{0}\alpha_{1}\ldots \alpha_{a}\ldots \alpha_{d}$ are the scaling factor according to the Tensor Train cores, $\alpha_{G}$ is scaling factor based on the reconstructed real weight and *f* stands for the filters’ index in the weight T where $T_{f}\in T$, $\alpha_{G_{f}}\in \alpha_{G}$. The Tensor Train cores are fixed after the training, thus we consider the α values are fixed as well, which means it would not require the L1-Norm mean operation in FPGA implementation. Focal Loss can be used as the supervision to alleviate the effect of class imbalance. However, it only supports the standard label of the [0,1] category. In order to enable the Focal Loss to train successfully on the joint model, autonomous quality focal loss is proposed, which optimizes the traditional focal loss in two parts: $-log\left(p_{c}\right)\to -\left(\left(1-y\right)log\left(1-\sigma \right)+ylog\left(\sigma \right)\right)$ and ${\left(1-p_{c}\right)}^{\gamma }\to {\left|y-\sigma \right|}^{\beta }\;\;\beta \geq 0$. The estimation σ denotes the output of the sigmoid operators [6,42]. The modulating factor ${\left|y-\sigma \right|}^{\beta }$ means the non-negative absolute distance between *y* and σ. When the estimation is accurate, σ will be close to *y*, the loss is down-weighted. Thus, we draw less attention to the simple example. Conversely, the distance ${\left|y-\sigma \right|}^{\beta }$ hard example produced will increase, attracting more attention to learn. Our final loss is defined as: $Loss={-|y-\sigma |}^{\beta }\left(\left(1-y\right)log\left(1-\sigma \right)+ylog\left(\sigma \right)\right),\beta \geq 0$. In addition, manually setting β to a fixed value has its drawback because both a high value of β and a low value β lead to an undesirable result [2]. Inspired by automated Focal Loss [2], we design a dynamically adjusted β to fit our model convergence during the training process. The value of β should be large enough to allow the network to focus on hard samples at the beginning, then the value of β should be lower to prevent decreasing gradients. Here, we apply $1-\hat{p_{c}}$ and bias *b* to numerically alter β. Therefore, the dynamic β is defined:

(9) $$\begin{array}{cc} \; & \beta =-log(1-\hat{p_{c}})-b\\ \; & where\;p_{c}=\left\{\begin{array}{cc} \sigma & \mathrm{if}\;y=1\\ 1-\sigma & \mathrm{if}\;y=others \end{array}\right. \end{array}\;$$

*p* represents the expected probability when a sample is predicted correctly. Furthermore, the $\hat{p}$ is roughly equivalent to the mean over *p* in one training batch, and we use $\hat{p}=0.95*{\hat{p}}_{old}+0.05*{\hat{p}}_{new}$ here to smooth the training process. After that, we reduce the frequency of change in β to train our model effectively, and the threshold for change is set to 0.05:

(10) $$\begin{array}{c} \beta_{i+1}=\left\{\begin{array}{cc} \beta_{i} & \mathrm{if}\;\beta_{i}-\beta_{i+1}<0.05\\ \beta_{i+1} & \mathrm{if}\;\beta_{i}-\beta_{i+1}\geq 0.05 \end{array}\right. \end{array}\;$$

where *i* represents the iteration.

## 4. Overall Hardware Architecture of ABFLMC-YOLOV5

**Figure 3:**
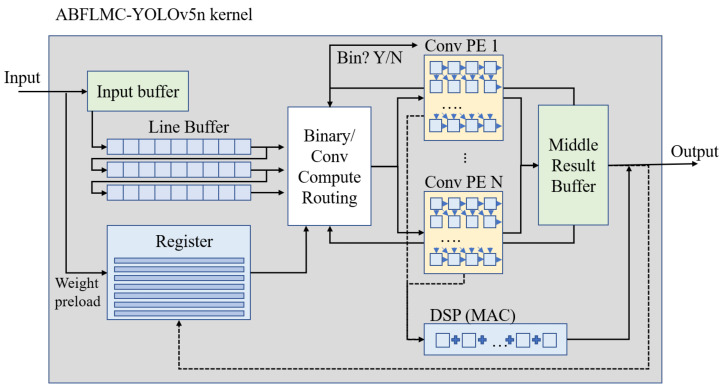
Overall hardware architecture of the implementation.

Figure 3 shows our proposed implementation of the FPGA architecture for ABFLMC-YOLOV5. The architecture consists of a computation kernel, registers, and BRAM. Our model presents a higher compression rate resulting in a relatively friendly environment for hardware implementation. Our core weight model size has only 1.39 MB in VOC dataset, which is one-fifth of the size compared to YOLOv5s. This allows fast-speed access from BRAM to become applicable. The PE (processing element) cluster is designed to address a large amount of parallel multiplication and accumulation within the convolution computation. A series of DSPs is implemented as a multiplied cumulative (MAC) system that performs the computation. Attributed to the ABFLMC-YOLOV5 algorithm, the parameters of our model were reduced to a certain level, which benefited from the scarcity of resources that is intrinsic for most low-power computing units. In Figure 3, the routing component computed by convolution is the core of the whole inference computation, which is responsible for running the overall algorithm. Schedule the timing and order of each computation and sends a control signal to access the required data. With our compressed model, the computation complexity for each layer presents an advantage when applied to a pipelining platform with sufficient hardware resources.

## 5. Experiment and Results

### 5.1. Experiment Setup

Our Yolov5n-based ABFLMC model performs the ABFLMC compression algorithm and the tensor train compression algorithm to shrink the model and simultaneously maintain accuracy. To demonstrate the effectiveness of autonomous quality focal loss in our model, we designed an ablation study based on the VOC dataset containing the Yolov5n model benchmark, the benchmark model with quality focal loss where γ=1.5 and Tensor Train compression, and our ABFLMC model, herein we set the dynamic bias β: b=1.0,1.4,1.5 separately, while keeping the other hyperparameters constant. Specifically, we set the epoch at 300, use rank = 16 to decompose our tensor to balance the size and precision of the model, and apply SGD optimizer with an initial learning rate of 0.01. Finally, our overall experiment is based on the PyTorch framework, and we use an Intel i9-9920X + RTX3090 PC as our hardware platform.

### 5.2. Ablation Study and Comparison with State of Art Models

As shown in Table 1, we use $mAP_{50:95}$, $mAP_{50}$ and the number of parameters to evaluate the performance of the model. Obviously, compared to the original model, the size of other models after compressing the tensor train decreases from 1.79 M to 1.39 M in the VOC Dataset. In terms of accuracy, our ABFLMC model with autonomous quality focal loss performs better than the traditional Focal Loss-Tensor Train (FL-TT) model. When the bias is b=1.4, our ABFLMC method achieves the best $mAP_{50:95}$ in 33.9 and $mAP_{50}$ in 62.1. Therefore, the proposed autonomous quality focal loss architecture efficiently enhances the basic FL-TT model. Besides, Table 1 also reveals that the higher *b* could shrink the difference between the hard samples and easy samples, resulting in the rapid mAP drop under the higher IOU threshold. However, the higher *b* is helpful for the lower IOU threshold to achieve higher performance according to its 0.1 drops of $mAP_{50}$ between b=1.4 and 1.5. In contrast, lower *b* is more efficient in keeping mAP below the higher IOU threshold because it can enlarge the discrimination by sampling. The model keeps the similar performance of $mAP_{0.5:0.95}$ between b=1.0 and 1.4. Furthermore, Table 2 shows that our ABFLMC model with b=1.4 achieves a competitive result compared to other state-of-the-art light-weight models. Our model achieves the smallest size with an acceptable accuracy drop in the comparison VOC dataset based. For the COCO dataset, although the $mAP_{50}$ of our ABFLMC model is 6 percent lower than the latest lightweight model yolov5n, our size is 22.1% less and computational complexity is 0.1 GFLOPs lower than the yolov5n. Specifically, our ABFLMC model reaches $mAP_{50}$ of 28.1 under VisDrone Dataset, which is 2.2× higher than yolov5n while keeping the light-weight design. This comparison also reveals that our ABFLMC design achieves the advantage under the large scene and small target dataset. Therefore, our model definitely has a better trade-off between accuracy and model size.

### 5.3. Hardware Evaluation

The hardware implementation environment is evaluated by the development evaluation board Ultrascale+ KCU116 on the XCKU5P FPGA from Xilinx. By reducing the size of the tensors and parameters with binarization, the model can be stored in the on-board storage (BRAM, regs) without using a DDR4 module. The results of the evaluation are listed in Table 3 for comparison and analysis. For the validation process of the ABFLMC model, a series of convolution computations have been applied. Thus, the process element cluster (PE) has been modified to adapt the convolution computation to speed up. With the binary computation, it only takes a small portion of computing resources compared to Float32. As a result, a mux function has been deployed in the routing of the computation in order to optimize the usage of PE speeding up the XOR computation with fewer registers and DSPs. The foremost factor affecting the on-chip power consumption will be the utilization of the Look-up Table (LUT) and Flip-Flops (FF) resources.

The differential function can define LUT as a register to store the active data or as a logic gate that fulfills the arithmetic requirement. In our design, LUT resources have been deployed about 51.3% for the VOC dataset and 55.56% for the COCO. The flip-flops usually work as a recording state; in most cases, they will be deployed as shared registers or high-speed buffering for the calculation. Due to the high compression rate of our model, the model size is available for pipelining design. FF utilization is approximately 40.2% for the VOC dataset and 41.2% for the COCO dataset.

The BRAM is used for storing compressed binary weight tensor cores and some float32 offset. BRAM utilization reaches 63.5% on VOC 07 + 12 and on the COCO Dataset. A dedicated MAC (Multiply Accumulate) unit has been implemented to speed up the convolution computing, which consists of multiple DSPs. In convolution computing, the addition operation can easily cause a delay due to a position replacement issue. However, DSP can be formed for a high-speed accumulator for a specific purpose to overcome the bottleneck of the computation process. Our DSP deployment shows 31.2% and 31.6% on each dataset. In Table 3, the power consumption is measured in two parts: on-chip power and off-chip power. We calculated the on-chip power for the FPGA ICs and included all peripheral devices on the board. The overall power consumption is presented in Table 3. The VOC Dataset reaches the power consumption of 6.12 W, and the GOPS reaches 135.5. The overall power consumption of the COCO Dataset is 6.33 W, and the GOPS reaches 129.4.

The state-of-the-art Yolov5 hardware comparison is presented in Table 4. However, most of the usage and result is on a different platform or did not mention in the article. We can only compare them in specific situations. With ABFLMC-YOLOv5n works, contributing to the auto focal loss and BNN quantization reducing a huge amount of the register resource utilization, the FPGA evaluation deployment can reach 6.33 W of power usage, which is only 39.3% of the TT-Yolov5s model under the COCO dataset and significantly lower than other high performance platforms. It is also difficult to monitor the power consumption of the whole operation on a mobile phone due to all the other high power-consuming parts besides the CPU (e.g., Screen, Camera). The mobile phone platform has the strength of mobility, but sacrifices the processing speed. Usually, the mobile SoC has dedicated compute units serving a specific function, only applying the general purpose compute units is quite inefficient. SVM simulation could be a possible way to enhance SoC performance according to Helali1’s work [49].

## 6. Conclusions

This article states a novel ABFLMC object detection model that combines efficient autonomous quality focal loss and compression of the tensor train with the acceleration of FPGA hardware. Our ABFLMC model achieves 33.9 in $mAP_{50:95}$ and 62.1 in $mAP_{50}$ with model size 1.79M and computational complexity 4.2 GFLOPs in the VOC Dataset, 22.8 in $mAP_{50:95}$ and 40.0 in $mAP_{50}$ with model size 1.48M and computational complexity 4.4 GFLOPs in the COCO Dataset, and 14.3 in $mAP_{50:95}$ and 28.1 in $mAP_{50}$ with model size 1.38M and computational complexity 4.1 GFLOPs in the VisDrone Dataset. Meanwhile, our ABFLMC method also benefits the computational efficiency of hardware implementation. In the VOC 7 + 12 dataset, the throughput reaches 135.5 GOPS while the power usage remains 6.12 W. On the COCO dataset, we present 129.4 GOPS with 6.33 W. The compression ratio of the model and the reduced number of operations give high flexibility for edge computing and other low-power applications. Moreover, the TT decomposition and BNN method still have the drawback of a drop in accuracy. Our architecture still has the potential to be improved in the future such as utilizing a lighter structure or more efficient BNN method to reduce computational complexity while maintaining more competitive accuracy.

## Figures and Tables

**Table 1:** Ablation Study under Different Bias in VOC.

Dataset	Model	${mAP}_{50:95}$	${mAP}_{50}$	# Paras
VOC 07 + 12	Original (Yolov5n)	45.4	72.5	1.79 M
	FL-TT (γ=1.5)	33.1	61.0	1.39 M
	ABFLMC (b=1.0)	33.9	61.7	
	ABFLMC (b=1.4)	33.9	62.1	
	ABFLMC (b=1.5)	33.6	62.0	

**Table 2:** Performance Comparison Across Models and Datasets [9,30,43].

Dataset	Models	Input Size	Backbone	${mAP}_{50:95}$	${mAP}_{50}$	# Parameters (M)	GFLOPs
VOC 07 + 12	Faster R-CNN [7]	600	VGG	-	73.2	134.7	-
	Faster R-CNN [7]	600	ResNet-101	-	76.4	-	-
	R-FCN [17]	600	ResNet-101	-	79.5	50.9	-
	SSD300 [3]	300	VGG	-	75.8	26.3	-
	DSSD321 [44]	321	ResNet-101	-	78.6	>52.8	-
	GRP-DSOD320 [43]	320	DS/64-192-48-1	-	78.7	14.2	-
	YOLOv5s [9]	640	-	51.9	78.4	7.11	16.5
	TT-YOLOv5s(rank 16) [9]	640	-	48.8	76.9	4.74	18.4
	MobileNetv2-Yolov5s	640	-	50.27	76.8	4.6	10.0
	ABFLMC-YOLOv5n	640	-	33.9	62.1	1.39	4.2
COCO	CenterNet-DLA [45]	512	DLA34	39.2	57.1	16.9	52.58
	CornerNet-Squeeze [46]	511	-	34.4	-	31.77	150.15
	SSD [3]	300	VGG16	25.1	43.1	26.29	62.8
	MobileNetv1-SSDLite [27]	300	MobileNetv1	22.2	-	4.31	2.30
	MobileNetv1-SSDLite [27]	300	MobileNetv2	22.1	-	3.38	1.36
	Tiny-DSOD [29]	300	-	23.2	40.4	1.15	1.12
	YOLOV4 [24]	320	CSPDarknet53	38.2	57.3	64.36	35.5
	YOLO-Lite [28]	224	-	12.26	-	0.6	1.0
	YOLOV3-tiny [47]	320	Tiny Darknet	14	29	8.85	3.3
	YOLOV4-tiny [24]	320	Tiny Darknet	-	40.2	6.06	4.11
	YOLObile [30]	320	CSPDarknet53	31.6	49	4.59	3.95
	YOLOv5s [48]	640	-	37.2	56.0	7.2	16.5
	TT-YOLOv5s (rank 16) [9]	640	-	34.2	54.6	4.9	18.9
	YOLOv5n [48]	640	-	28.4	46.0	1.9	4.5
	ABFLMC-YOLOv5n	640	-	22.8	40.0	1.48	4.4
VisDrone	YOLOv5n [48]	640	-	12.9	25.9	1.78	4.2
	ABFLMC-YOLOv5n	640	-	14.3	28.1	1.38	4.1

**Table 3:** Hardware evaluation in different datasets.

Hardware Evaluations	VOC 07 + 12	COCO	VOC 07 + 12	COCO
Model Name	ABFLMC-Yolov5n		TT-Yolov5s [9]	
Param M	1.39	1.48	4.74	4.9
LUT	111,233	120,533	182,022	187,022
LUT Utilization (%)	51.3	55.6	83.9	86.2
FF	174,210	178,720	123,098	143,728
FF Utilization (%)	40.2	41.2	28.4	33.1
BRAM (MB)	305	305	220	235
BRAM Utilization (%)	63.5	63.5	45.8	49
DSP	569	577	1321	1351
DSP Utilization (%)	31.2	31.6	72.4	74.1
GOPS	135.5	129.4	42.6	34.2
Power (W)	6.12	6.33	15.2	16.1

**Table 4:** Hardware Comparison in different platforms within COCO dataset.

Models	Dataset	Platform	GFLOPs	Power (W)
ABFLMC-YOLOv5n (Ours)	COCO	Intel i9-9920X + RTX3090	4.4	200
ABFLMC-YOLOv5n (Ours)	COCO	Ultrascale + KCU116 FPGA	4.4	6.33
TT-YOLOv5s (rank16) [9]	COCO	Ultrascale + KCU116 FPGA	18.9	16.1
YOLObile [30]	COCO	Qualcomm Snapdragon 865	3.95	5 *
YOLOv5n [48]	COCO	na	4.5	na
REQ-YOLO [50]	VOC 07 + 12	ADM-7V3 FPGA	na	21

* The power is not mentioned in the article. We estimated it only based on the theoretical TDP of the SoC.

## Data Availability

The data presented in this study are available on request from the corresponding author upon reasonable request.
